# The Hidden Benefits of Septal Myectomy: The Journey Toward a More Holistic Approach∗

**DOI:** 10.1016/j.jacadv.2023.100762

**Published:** 2023-12-12

**Authors:** Dermot Phelan, John Symanski

**Affiliations:** Sanger Heart and Vascular Institute, Atrium Health, Charlotte, North Carolina, USA

**Keywords:** hypertrophic cardiomyopathy, left ventricular outflow tract obstruction, septal myectomy, sexual health

**Figure:**
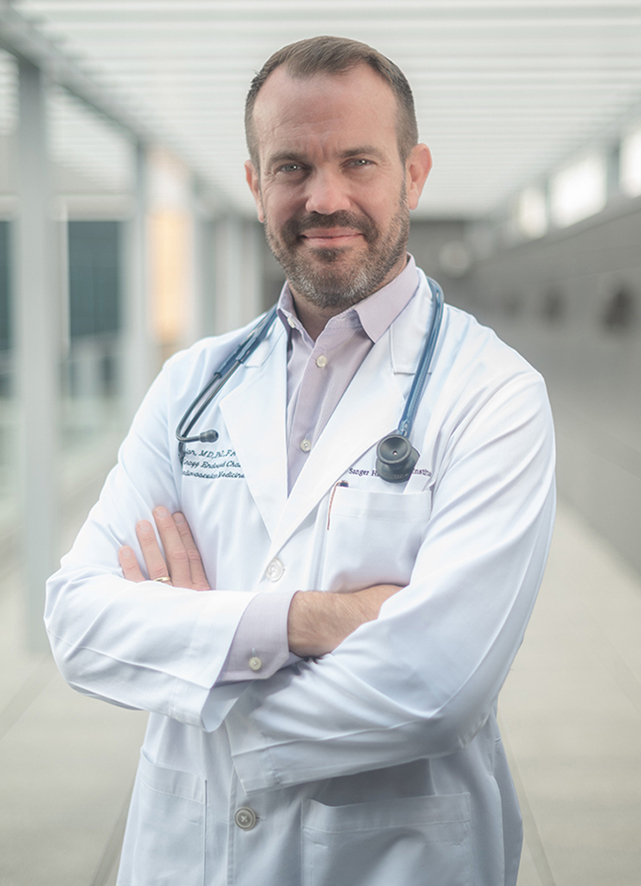

*“Sexual health is a state of physical, emotional, mental, and social well-being in relation to sexuality; it is not merely the absence of disease, dysfunction, or infirmity.”*

—World Health Organization

Hypertrophic cardiomyopathy (HCM) is a complex condition characterized by dynamic symptoms that can significantly impact the daily lives of affected individuals. While septal myectomy has long been established as an effective treatment for alleviating the physical symptoms associated with obstructive HCM, there has been limited prospective data regarding the broader functional outcomes of this procedure, particularly in terms of sexual health. The World Health Organization recognizes that sexual wellness plays a vital role in one’s overall health and well-being and that sexual health goes beyond the absence of disease or dysfunction, encompassing physical, emotional, mental, and social dimensions of an individual's life. And yet, health-care providers have routinely overlooked sexual health assessment in the history and physical examination. The study by Nguyen et al^1^ in this issue of *JACC: Advances*, conducted at the Mayo Clinic, sought to bridge this knowledge gap by quantifying the changes in patients' quality of life (QOL) and prevalence of sexual dysfunction before and after septal myectomy for obstructive HCM.^1^ The Mayo group has an extensive experience and proven record of successful outcomes with this operation, and they are to be congratulated for addressing this vitally important and neglected issue.

Conducted between January 2018 and October 2019, QOL measures were tabulated using the Patient-Reported Outcomes Measurement Information System (PROMIS) incorporating questions from the Information Index of Erectile Function (IIEF-15; score range 1-30) and the Female Sexual Function Index (score range 2-36) to quantify degrees of sexual dysfunction in men and women, respectively. Survey questions relating to urinary symptoms were derived from the International Prostate Symptoms Score, a validated tool for both men and women. The principal findings of this study were: 1) highly statistically significant improvements in *physical* PROMIS scores following myectomy in both females (38.3 ± 7.9 vs 49.1 ± 7.8; *P* < 0.001) and males (41.4 ± 7.9 vs 51.2 ± 9.7; *P* < 0.001), as well as improvements in *mental health* PROMIS scores for females (45.5 vs 53.1; *P* < 0.001) and males (48.8 ± 8.2 vs 53.6 ± 8.4; *P* < 0.001); 2) improvements in cumulative Female Sexual Function Index score (23.8 vs 29.1) and specific domains of sexual function in females; and 3) no change in IIEF score among all male respondents (median presurgery and postsurgery score of 23 indicating mild erectile dysfunction). A subanalysis showed significant improvement in IIEF scores among younger males (age 22-55 years); however, males over age 55 reported worsening of erectile dysfunction postmyectomy.

Improvements in sexual function and QOL measures were attributed to alleviation of left ventricular outflow tract gradients (mean postbypass left ventricular outflow tract gradient 2 ± 3.2 mm Hg) and reduction of preoperative medications used to manage obstructive symptoms. Unfortunately, no details regarding preoperative and postoperative use of beta blockers, disopyramide, or diuretics were provided. While intuitive and plausible, worsening of erectile dysfunction observed postoperatively among older males would not support this contention.

Several limitations and potential biases of the Mayo study warrant consideration. The authors point out that the complete survey response rate of 57.4% excluded a significant segment of the study participants. While baseline characteristics of survey responders and nonresponders were not substantially different, there may be other factors that lead to an unwillingness to complete participation such as a perceived failure to meet surgical outcome expectations or hesitancy in responding to subsequent questions due to persistent symptoms. It is important to note that individuals referred for septal myectomy constitute a highly symptomatic subgroup of HCM patients who have been unresponsive or intolerant to standard medical treatments. One would anticipate positive survey responses from patients in this group, as their longstanding symptoms have likely significantly improved with the emotional relief of overcoming a previously feared open-heart operation. The lack of a control group also limits interpretation of the study findings. It has been well documented that sham procedures, as explored in other interventional trials (chronic coronary syndromes, for example), can exert powerful placebo effects, leading to reported improvements in symptoms, QOL, and exercise capacity.^2^

What resources are currently available to assess concerns related to QOL? Kansas City Cardiomyopathy Questionnaire-Clinical Summary Scores (KCCQ-CSS) following septal myectomy were recently detailed by Desai et al^3^ in the SPIRIT-HCM study from the Cleveland Clinic, another excellent high-volume surgical center. This analysis included 173 patients with obstructive HCM who underwent septal myectomy with 79% of enrolled patients completing a median follow-up of 14 months. A ≥5-point increase in KCCQ score was observed in 92% of patients after surgery with 80% experiencing a >20-point gain. Only 8% of patients did not improve their KCCQ scores. An important outstanding question is whether a similar degree of improvement in QOL scores and sexual dysfunction would be observed among those who achieve gradient reduction and symptom benefit with conventional medical therapies or alcohol septal ablation procedures.

Tremendous excitement has recently been generated by the novel myosin-modulator therapies. The pivotal phase 3, EXPLORER-HCM trial^4^ included patient-reported outcomes using KCCQ and Hypertrophic Cardiomyopathy Symptom Questionnaire Shortness-of-Breath scores among 123 patients treated with mavacamten and 128 placebo controls. All patients were receiving standing medical therapy with beta-blocker and/or nondihydropyridine calcium channel blockers. Those on mavacamten exhibited significant improvement in KCCQ score (9.1, 5.5-12.7) and reduction in Hypertrophic Cardiomyopathy Symptom Questionnaire Shortness-of-Breath score (−1.8, −2.4 to −1.2) [*P* < 0.0001]. Notably, the observed improvement in KCCQ score was several-fold higher than that observed in recent heart failure drug trials.^5^ In a separately published prespecified health status subanalysis of EXPLORER-HCM, the proportion of patients with a very large gain in KCCQ overall score (≥20 points) was 36% (33/92) in the mavacamten group vs 15% (13/88) in the placebo group with an estimated absolute difference of 21% and number needed to treat of 5.^6^ KCCQ scores also served as a secondary end point in the VALOR-HCM trial.^7^ In this phase 3 double-blind study, 112 patients who met guideline criteria for septal reduction therapy were randomized to placebo or mavacamten 5 mg daily, titrated up to 15 mg. After 16 weeks of treatment, 43 of 56 placebo patients (76.8%), but only 10 of 56 mavacamten patients (17.9%) continued to meet guideline criteria or underwent septal reduction therapy. KCCQ score improved by 9.4 points in the mavacamten-treated patients (*P* < 0.001). While we laud efforts in recent studies to focus on QOL issues, exploring the effect of these novel therapeutics on sexual health must be encouraged as an important next step.

How should the findings of the present study be incorporated into the management of the HCM patient population? Continued refinement of the tools designed to measure QOL and sexual wellness are warranted making their adoption into clinical practice simple, recognizable, and understandable. It is important in the HCM population to recognize complex and inter-related factors that impede physical, emotional, and sexual wellness. The burden of harboring a chronic, heritable condition that incurs risks to self, affected family members, and potential offspring cannot be minimized. Financial burdens associated with lifelong medical care and potential need for life-altering therapies including implantable cardioverter defibrillator implantation, septal reduction procedures, and cardiac transplantation are additional sources of ongoing concern for many HCM patients. Future research must consider the broader implications of medical interventions, assessing their effects not only on the physical well-being of patients but also on their emotional, mental, and social dimensions. This study underscores the interconnectedness of sexual health, QOL, and physical well-being. Striving for a comprehensive, integrated, and holistic approach to HCM care must be a shared objective for all health-care providers.

## Funding support and author disclosures

The authors have reported that they have no relationships relevant to the contents of this paper to disclose.
